# Review of Prosthetics & Orthotics Needs for 21st Century – Vision for 2025

**DOI:** 10.33137/cpoj.v4i2.37113

**Published:** 2021-09-21

**Authors:** S Zahedi

**Affiliations:** Blatchford Group, Unit D Antura, Bond Close, Basingstoke, RG24 8PZ, United Kingdom.

**Keywords:** Prosthetic, Systems, Future integration, Health Economics Effectiveness, Rehabilitation, Assistive devices, Technology

## Abstract

What would we do, if only we had the power to go back?! The best way to consider this is to align and join all the known dots. To think of Prosthetics and Orthotics (P&O) as a system holistically centred around care of the user, identifying all their needs continuously, in their environment and in their lifestyle. This could produce a new value proposition for all multi-disciplinary team members by generating patient-centred therapeutic benefits and clinical outcomes that align all stakeholders in P&O towards using a common narrative, which makes decisions based on data. In this case, data is the outcome, using Standards and Instruments which are validated (e.g. www.amprom.uk) to quantify questions such as: “Have we reduce risk of falls?”, “Have we reduced risk of tissue injury?”, “Have we reduced risk of low back pain?”, “Have we reduced long term risk of osteoarthritis?”, etc. If we have, we are assured this will benefit the comfort and confidence for the user. We can have confidence in rehabilitation measured by improved stability and increased activity, and other measures which enable the accurate classification of products and services to match users. A prescription index, based on Outcomes, could, for example, be calculated by a formula which accounts for the percentage reduction in falls probability, a patient satisfaction score, a mobility score and a quality-of-life score, allowing practitioners to base their choices of treatment pathways and component selection. This paper provides both the context for and contributing factors that make the proposing of such an objective Prescription Index an interesting thing to consider when discussing Health Economics in P&O.

## THE CONTEXT: A GLOBAL CHALLENGE

In Lower limb Prosthetics, there are 10 million amputees worldwide.^1^ There are estimated 10,000 practitioners each dealing with 400 patients in a year. This then only allows the needs of 4 million amputees to be addressed, leaving 6 million without access to care. WHO Standards call for 4-5 professionals per million population. WHO's recent figures of the disabled population stated 2.4 billion people need rehabilitation, assistive technology and mobility solutions. Yet, the qualified groups of professionals in the P&O sector remain in the thousands, which is inadequate to meet the needs of the millions of patients needing care.

With the rise of poverty worldwide, there is a need to use the limited healthcare resources more efficiently. Validated and verified (health) economics tools, implemented within each country's budget guides for best utilisation of resources, along with the deployment of appropriate technology, can guide decision-making on products and services with assured outcomes.

## THE OPPORTUNITY: DIGITAL HEALTH

In prosthetics, socket comfort remains at the heart of the lower limb amputee rehabilitation prosthetic challenge. The interface with the residuum needs to be addressed based on real, objective science. This connection of socket to residual limb is, itself, a joint that dynamically moves and changes shape and volume due to skeletal bone movement inside muscles, tendon and skin soft tissue.^2,3^ Digital tools should be using algorithms developed from, and based upon, dynamic input from sensors at the interface with the residuum measuring shear and compression forces and simultaneously to convert this information to simulate the movement of this unique joint. Next, cancelling this movement perception by opposing actuating mechanisms, to create a perception of instantaneous rest (a direct skeletal fixation feel) in all dynamic conditions will bring deeper science to this last area of black magic.^4^

Data from smart liners with embedded sensors allow such objective measures to be collected. This enables both subjective and objective virtual assessment, with web-based accessibility allowing communication to an expert in any location around the globe to share knowledge and clinical experiences. This digital data is what is needed to create objective Prescription Indices to guide P&O decision-making. Tele-medicine (for initial assessment, virtual triage, and final follow up) combined with hand held scanners, 3D additive manufacture printers, mobile centres for fitting, fabrication at remote satellite centres, and drone deliveries are already a reality.^5,6^

**Figure 1:**
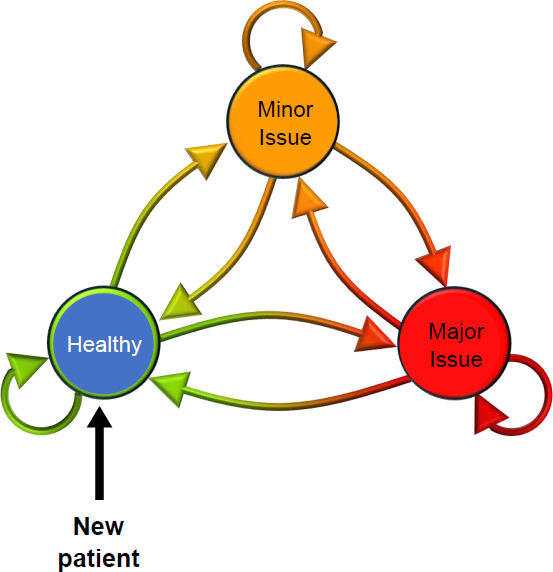
Systems Thinking: three health states needing different resources. (Diagram from Blatchford Institute)

**Figure 1** shows a Systems thinking model for continuous monitoring of patients that supports best function, creating rehabilitation pathways using local resources and allowing for experts' experiences to be accessed for addressing issues. Within this model, data collected can also be used to prevent costly tissue damage and to enable user participation in their rehabilitation. This is the collaborative way of addressing this major challenge. It is estimated over 50% of amputees need one socket or major adjustment per year at a treatment cost $10k, so working solutions to address this can result in considerable cost savings.^5,7,8^

## A FUNDAMENTAL REALITY: HEALTH ECONOMICS

Health Economics plays a daily role in making decisions in all areas of health care. The American Orthotics and Prosthetics Association (AOPA) understood this when engaging the RAND Corporation to carry out the Microprocessor Controlled Knee (MPK) project to produce a report for use in justification of equivalent models.^9^ This in turn set in motion the UK's National Health Service's MPK Policy and leads the narrative on health economics/cost-benefit/future business models in P&O that are linked to outcome measures.^10^ Based on evidence, these works are being used for policy making decisions and developing technology road maps. Some examples of the measures used to support decision making in the above process are:

### Quality Adjusted Life Year (QALY)

The QALY is a healthcare measure that takes into account both the quantity and quality of life. One QALY indicates one year of perfect health. Additional QALYs provided by a given intervention, B, compared to an existing treatment, A, is calculated by the difference in Utility scores for each intervention for a given year (determined from certain patient-reported outcome measures, such as EQ-5D-5L or SF36) multiplied by the number of years, over which the treatment is being considered.

### Financial cost

Though not a patient health consideration, inevitably the financial cost of a new, innovative intervention will always be considered. In particular, it will be compared to the cost of existing, alternative treatments and must be weighed against the potential for patient benefit.

### Incremental Cost-Effectiveness Ratio

Incremental Cost-Effective Ratio (ICER) is a means of factoring both patient benefit and fiscal burden into a single metric. It is calculated as the ratio of the difference financial cost between the new treatment and the existing one, to the QALYs added by the new treatment.


$$ICER=\frac{Cost_{B}-Cost_{A}}{time.(Utility_{B}-Utility_{A})}$$

**Figure 2:**
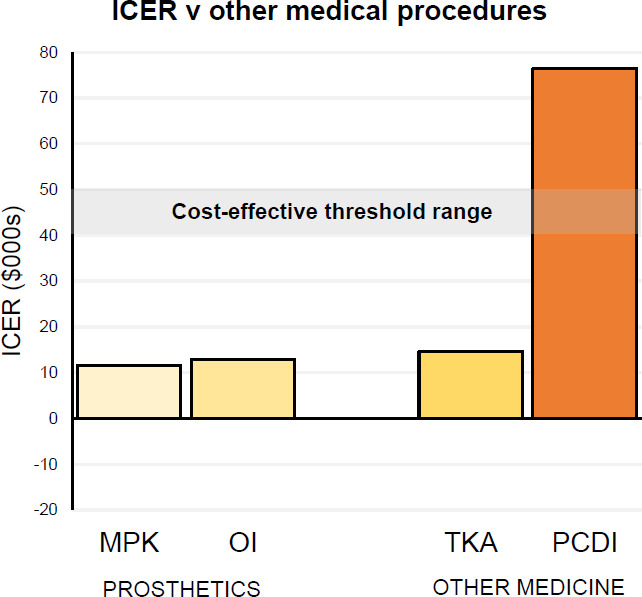
Incremental Cost-Effectiveness Ratio comparison of Microprocessor Prosthetic Knees (MPK), Osseointegration (OI), Total Knee angioplasty (TKA), and Prophylactic cardioverter defibrillator implantation (PCDI). All values in 2016 USD9,11. (Diagram from Blatchford Institute)

In the RAND study,^9^ ICER was used to do a cost effectiveness comparison of MPKs with other commonly funded medical procedures (**Figure 2**). Also added is data reported on another prosthetics intervention: Osseointegration^11^ (OI). Costs for OI have been converted from the originally reported 2016/17 Australian dollars to 2016 US dollars (1 AUD: 0.76 USD) to aid comparison with other reported data.^9^

## POTENTIAL FUTURE DISRUPTOR: OSSEOINTEGRATION

While there are tools and measures such as the QALY that can be used to objectively evaluate current technology and processes, new techniques such as OI have the potential to be very disruptive to the status quo. As work progresses on this, members of the Prosthetics Working group of the International Standards Organization (ISO) are in process of setting an ISO subgroup to look at fail safe mechanism requirement specifications, test methods and test loads for Osseointegration. The first subgroup workshop on this topic was organised with the Assessment Research Centre (ARC) at the University of Melbourne where it was demonstrated that a variety of failsafe mechanism designed by different organisations had been done so with little reference to structural safety load standards used in lower limb prosthetics. All manufacturers are now keen to work together to establish a common standard to reduce rate of mechanical failures and protect users. This will, in time, will lead to a common procedure for selection, standard surgical operating procedures and post-operative care that will reduce risk of implant failures.^12^ Once OI is an established rehabilitation pathway, it will open up a whole new dimension in System Thinking in lower limb (already envisioned by some of the organisation in upper limb), as the direct route to connectivity to physiological nerves and muscles is provided. This will also have an impact on the economics of prosthetic care throughout the lifetime of the patient.

## AN UNADDRESSED NEED: EDUCATION

Technology and Digital health at affordable prices needs to be the facilitator and robust science must replace the black magic upon which much of our current understanding of P&O is based. Furthermore, this must be done with the support of P&O educators. Currently most P&O educators are simply asking if their courses are fit for purpose – which is to support and maintain the status quo. It must be asked critically and answered with honesty:

- Will the graduates of 2025 have the right knowledge to meet the global challenges they will face?
- Will they know how to reduce the risk of tissue injury?
- Will they understand the effect of pressure, shear and moisture on the residual limb interface?
- Will they be able to use objective data? (e.g. by correctly reading and interpreting data provided by innovative measurement systems)
- Will they be able to use the results to make clinical, design and fit decisions?
- Will the P&O practitioner have the knowledge and skills to treat the socket to residuum interface as a joint and be able to manage the bone movement inside soft tissue?
- How will they integrate sensors in a new product (that can detect and adjust the interface device independently) into their practices in a way that is also economically viable?

All this, and more, becomes new material to be integrated into the curriculum if educators are to prepare the clinicians of the future for the technology of product and services together, which will be arriving as early as 3–5 years from now. In adopting them, these technologies need to be justified by health economics/cost-benefit/future business models in P&O and must be linked to outcome measures that are based on evidence. This is crucial for policy making decision and continued development of the technology road map.

**Figure 3:**
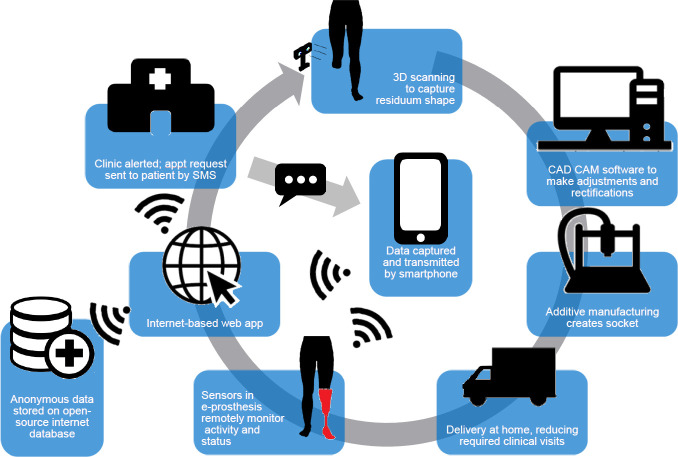
“Servitisation” model for Local/Satellite and central fabrication. Pilot study illustration from MovAid^13^

One way of describing this future model is a future of “Servitisation”, which is the integration of Product and Services together. Customised to individual need, it is made in a bespoke manner. An example of what this model could look like is shown in **Figure 3**.

**Figure 4:**
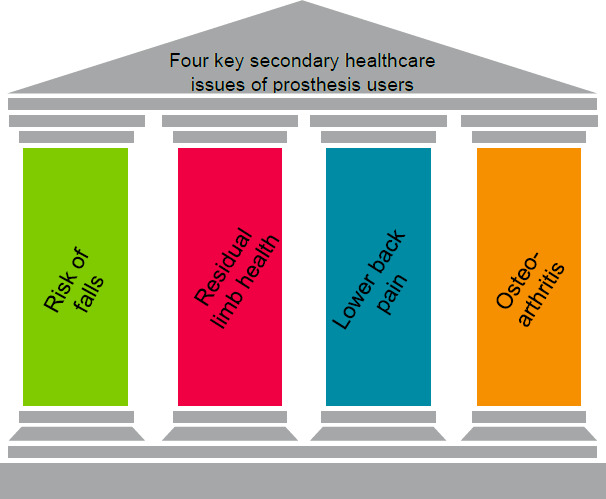
Educational Need for future “Servitisation” – A four Pillars Model for Lower Limb Prosthetics Using Validated Patient Reported Outcomes (Diagram from Blatchford Institute)

P&O educators have a role to play in including such new ways of structuring into what we know and what the data tells us. We can integrate this into existing health economics and care models such as (for example) a “Pillars of Health Care” approach. In lower limb prosthetics, the four Pillars for Heath Economic Evidence are reducing the risk of falls, tissue injury, lower back pain and osteoarthritis (**Figure 4**). Doing so requires not only new models and data, but also e-documentation, generating new data supporting the pillars of evidence based practice.

## A BLACK SWAN? ACCELERATION BY COVID

Industry moving towards a digital health cloud-based system is well on the way. The vision of 2020 has most recently been accelerated by the 2020-2021 global Covid-Virus Pandemic. In the UK, the system interface gateways are already in the advanced stage with NHSx (www.nhsx.nhs.uk) which is overseeing the digitization of the NHS in partnership with other healthcare providers building the required infrastructure.

Accelerated by Covid, investors are increasingly looking to opportunities with Medical technology and more specifically devices with apps, whose usage is becoming a core part of care. Virtual assessment in P&O services and online triage are going to stay in this New World and will be harnessed as part of cost-rationalisation. This is more necessary than ever as pressure of budgets on healthcare and the extra cost of advances in technology, compel all stakeholders to look to cut out waste, increase productivity and become leaner. The review of Prosthetic services is already taking place in UK, re mapping the provision for next 10 years. The global community response to the WHO GATE project^1^ expands the opportunity in their call for care of the world's 1billion disabled people and acknowledges that addressing mobility needs via Assistive Technology will become an even greater challenge, with millions more people with disability to be caused by Covid-19. There already exists an insufficient number of clinicians to cater to 60% of cases across the world,^1^ so there remains a challenge for all with respect to responding to this global need. The gap between developed and developing nations will become even wider. A corresponding rise of poverty rates will make achieving UN Sustainable Development Goals even harder. With increasing private investors ownership of P&O companies, longer term vision may suffer, but there is more potential for capital investment to respond to these challenges with viable solutions.

### Changes already underway

More recently, there has been a move by many suppliers to central fabrication and utilisation of 4th industrial revolution technology for sustainability of logistic supply chain and delivery improvement. Central Fabrication was the standard method of services some 40 years ago. With advances in engineering, the shift from a craft-based system to industrial systems, the creation of modular assembly Prostheses & Orthoses, in the last century there was a divergence and separation of product and services. The craft-based technician capable of fitting a patient became the clinical Prosthetists and Orthotists, who were trained and educated in universities and colleges resulting in the generation of degree-qualified education of P&O healthcare professionals, who have served our communities. The evolution of technology is now again converging product and services. This requires a review of current education and training. It must be asked: Is the current education a fit for future? Does it align with future requirement of Multi-Disciplinary Team care of disabled population? Does it meet industry and health care needs? Any suppliers who only provide services are finding it difficult to grow. The suppliers of P&O components are now developing products and technology where the designed and manufactured products must be directly fitted to patients, matched objectively to the user in shape, form, activity and lifestyle. The only thing that is not changing and remains certain is the how all these parameters will be changing.

The future holds customisation and bespoke devices which are made to measure to fit and with embedded technology. Devices that sense changes and automate the process of scheduling replacements and which will, eventually, automatically adjust themselves in response to change, are all part of the future of P&O care.

### Regulatory Evolution

With increasing globalisation and reliance on technical solutions, there is a need for safety, as well as policing against rogue players and the development of a level playing field. The emergence of evidence-based practice and the required evolution of validated and verified outcome measures are critical to protect the patient. Documentation of mitigation of risks and decision-making, as well as continuous monitoring, feeding continuous development is rapidly becoming a standard procedure across all medical industries. P&O will not be exempt. The emergence of the EU Medical Devices Regulations (MDR)^14^ replacing previous Directive in Europe, and its alignment with FDA in North America, will all be sitting on a platform of Medical Quality international standards. All stakeholders must be preparing for and expecting compliance. These changes will require clinicians, by law, to assess risk versus benefit and to make decisions based on previously collected and systematically reviewed objective evidence.

## CALL TO ACTION

### 1) Adoption of a “Pillars of Health” model, as described above

The pillars must be defined by and supported by evidence-based data supported criteria and must be economically justifiable. Those responsible for making this happen are all the authorities, organizations and persons in power in P&O sector

### 2) Making changes to P&O education

P&O Educators must be educating P&O students to be able to adopt digital tools and digital ways of thinking. Graduates must be able to make decisions based on evidence supported by data, where that data is comprised of Outcome, Standards and Instruments, that are validated, in order to quantify what has been done. This will ensure that all decisions are based on data and economic justification. They must also understand: how the data inform and support the pillars of healthcare, the relationship between economics, regulation and policy, and how each will be changed by the digitisation of healthcare. The educational outcome must be a practitioner that uses the same narrative across all of P&O sector. Finally, as technology and digital health's costs go down and become available at an affordable price, there is a need for a graduate who can act as a facilitator for future rehabilitation and robust science - a model that must replace the current craft model. This cannot be achieved without the support of P&O educators.

## DECLARATION OF CONFLICTING INTERESTS

I am an employee of Blatchford.

## SOURCES OF SUPPORT

There are no external financial support. Horizon 2020 EU grant 2014-2017
